# Cultural adaptation and validity evidence of the Student Nurse Stressor-15 (SNS-15) Scale for Brazil

**DOI:** 10.1590/0034-7167-2023-0356

**Published:** 2024-03-15

**Authors:** Agostinho Antônio Cruz Araújo, Simone de Godoy, Natália Maria Freitas e Silva Maia, Maria Eduarda Bonissoni Trevelin, Kelly Graziani Giacchero Vedana, Carmem Beatriz Neufeld, Neyson Pinheiro Freire, Carla Aparecida Arena Ventura, Patricia McAleer, Isabel Amélia Costa Mendes

**Affiliations:** I Universidade de São Paulo. Ribeirão Preto, São Paulo, Brazil; II Universidade Federal do Piauí. Teresina, Piauí, Brazil; III Conselho Federal de Enfermagem. Brasília, Distrito Federal, Brazil; IV NetwellCASALA, Dundalk Institute of Technology. Dundalk, County Louth, Ireland

**Keywords:** Education, Nursing, Stress, Psychological, Students, Nursing, Validation Study, Psychometrics, Educación en Enfermería, Estrés Psicológico, Estudiantes de Enfermería, Estudio de Validación, Psicometría, Educação em Enfermagem, Estresse Psicológico, Estudantes de Enfermagem, Estudo de Validação, Psicometria

## Abstract

**Objectives:**

to carry out the cultural adaptation and evaluation of validity evidence of the Student Nurse Stressor-15 (SNS-15) Scale for use in Brazil.

**Methods:**

psychometric study, conducted from the stages of translation, synthesis, back-translation, review by a committee of seven experts, pre-test and evaluation of measurement properties with 32 and 238 nursing students, respectively. Descriptive statistics, Exploratory Factor Analysis (EFA), and Confirmatory Factor Analysis (CFA) were performed. The reliability of the instrument was estimated using McDonald’s Omega (ω).

**Results:**

EFA subsidized the distribution of the fifteen SNS-15 items into four factors. Using AFC, satisfactory fit indices were achieved (Comparative Fit Index = 0.94; Tucker-Lewis Index = 0.93; Root Mean Square Error of Approximation = 0.06; Standardized Root Mean Square Residual = 0.16) and ω = 0.86.

**Conclusions:**

the Brazilian version of the SNS-15 presents evidence that confirms its validity and reliability.

## RESUMO

**Objetivos:**

realizar a adaptação cultural e a avaliação das evidências de validade da *Student Nurse Stressor-15 (SNS-15) Scale* para uso no Brasil.

**Métodos:**

estudo psicométrico, conduzido a partir das etapas de tradução, síntese, retrotradução, revisão por um comitê de sete especialistas, pré-teste e avaliação das propriedades de medida com 32 e 238 estudantes de enfermagem, respectivamente. Realizou-se estatística descritiva, Análise Fatorial Exploratória (AFE), Análise Fatorial Confirmatória (AFC). Estimou-se a confiabilidade do instrumento através do Ômega de McDonald’s (ω).

**Resultados:**

a AFE subsidiou a distribuição dos quinze itens da SNS-15 em quatro fatores. Mediante AFC, alcançou-se índices satisfatórios de ajuste (*Comparative Fit Index* = 0,94; *Tucker-Lewis Index* = 0,93; *Root Mean Square Error of Approximation* = 0,06; *Standardized Root Mean Square Residual* = 0,16) e ω = 0,86.

**Conclusões:**

a versão brasileira da SNS-15 apresenta evidências que confirmam sua validade e confiabilidade.

## RESUMEN

**Objetivos:**

realizar adaptación cultural y evaluación de evidencia de validez de la *Student Nurse Stressor-15 (SNS-15) Scale* para su uso en Brasil.

**Métodos:**

estudio psicométrico, realizado desde las etapas de traducción, síntesis, retrotraducción, revisión por un comité de siete expertos, pretest y evaluación de propiedades de medición con 32 y 238 estudiantes de enfermería, respectivamente. Se realizó estadística descriptiva, Análisis Factorial Exploratorio (AFE), Análisis Factorial Confirmatorio (AFC). La confiabilidad del instrumento se estimó mediante el Omega de McDonald (ω).

**Resultados:**

AFE subvencionó la distribución de los quince ítems del SNS-15 en cuatro factores. A través de la AFC se lograron índices de ajuste satisfactorios (*Comparative Fit Index* = 0,94; *Tucker-Lewis Index* = 0,93; *Root Mean Square Error of Approximation* = 0,06; *Standardized Root Mean Square Residual* = 0,16) y ω = 0,86.

**Conclusiones:**

la versión brasileña del SNS-15 presenta evidencias que confirman su validez y confiabilidad.

## INTRODUCTION

Stress is an adaptive response caused by situations of mental or emotional pressure. The reaction to stress is subjective, that is, each person has a different coping capacity, considering genetic particularities, aspects related to life history, personality, as well as social and economic context^(1)^. In turn, a stressor is defined as a situation or event that triggers stress, causing a subjective state of physical or mental tension^(2)^.

In general, stress affects the musculoskeletal, respiratory, cardiovascular, endocrine, gastrointestinal, nervous and reproductive systems and can impair decision-making^(3-5)^. In relation to social interaction, the response to stress varies according to the particularities of the stressor and the individual, and may adopt a pro-social stance, avoidance or risk behaviors^(6-7)^.

The survey entitled “Stress in AmericaTM 2022”, conducted by The Harris Poll on behalf of the American Psychological Association (APA), shows that the population of the United States of America (USA) is vulnerable to stressors that are beyond their control i.e., aspects inherent to society, such as the repercussions of COVID-19, racial injustice and political division^(8)^. Limitations in controlling stressors also occur in the educational environment, since studies report that obtaining a university education is considered stressful^(9-10)^. Specifically in nursing, stress can be manifested more intensely during clinical learning^(11-15)^, a fundamental stage in the development of skills and abilities and, in the future, for the performance of the profession^(13,16)^.

As a way of identifying the stressors experienced by nursing students during their clinical activities with the elderly population, researchers from Ireland and Australia developed the Student Nurse Stressor-15 (SNS-15) Scale. This scale establishes 15 potentially stressful situations, in order to identify them and, thus, promote prevention approaches to minimize the negative impact in the short and long term on the student’s training process and, consequently, promote better patient care. The SNS-15 achieved satisfactory values in the following indices: Comparative Fit Index (CFI): 0.93, Tucker-Lewis Index (TLI): 0.92 and Root Mean Square Error of Approximation (RMSEA): 0.06^(17)^; which demonstrate the validity of the original version.

Therefore, with the cultural adaptation and evaluation of evidence of validity of the Student Nurse Stressor-15 (SNS-15) Scale, it is expected that in Brazil this instrument will help identify stressors experienced by nursing students in clinical practice, in order to support the development of institutional interventions to reduce academic stress, underpin the teaching-learning process and increase student retention. Investing in student nurses to retain them in their courses is imperative, especially considering the shortage of nurses in workplaces globally^(18-20)^. Furthermore, when considering the evidence of the validity of the SNS-15 construct and the mobilization of other countries (China and Turkey) in conducting the cultural adaptation process, it is essential to culturally adapt and evaluate the evidence of validity of this scale in a Brazilian context.

## OBJECTIVES

To carry out the cultural adaptation and evaluation of validity evidence of the Student Nurse Stressor-15 (SNS-15) Scale for use in Brazil.

## METHODS

### Ethical aspects

The study was approved by the Research Ethics Committee of the Ribeirão Preto College of Nursing at the University of São Paulo. All participants signed the Free and Informed Consent Form (TCLE). Furthermore, the research offered minimal risks, such as discomfort and fatigue related to the use of time to complete the instrument. In view of this, it should be noted that the response deadline was made more flexible. In addition, confidentiality was guaranteed by using the information only for the purposes of the study.

### Study design, setting, and period

Psychometric study for cultural adaptation and evaluation of validity evidence of the Brazilian version of the Student Nurse Stressor-15 (SNS-15) Scale. The research was carried out in two stages, namely: 1) Cultural adaptation, which comprised the stages of translation, synthesis, back-translation, review by a committee of experts, and pre-test with nursing students^(21)^; 2) Assessment of measurement properties by another group of nursing students.

The study was conducted between October 2021 and November 2022 at a public state university, located in São Paulo, Brazil. This Higher Education Institution (HEI) offers two undergraduate courses in Nursing: Bachelor’s Degree in Nursing and Bachelor’s Degree and Degree in Nursing.

### Study protocol

#### Translation

The Student Nurse Stressor-15 (SNS-15) Scale is composed of 15 items, distributed in two domains: Knowledge and workload (1, 2, 3, 4, 5, 6, 7, 14, 15); and Resources (8, 9, 10, 11, 12, 13). Regarding the instrument score, the answers are in a five-point Likert format, as follows: 1 - Very Stressed, 2 - Stressed, 3 - Neutral, 4 - Moderately Stressed and 5 - Not Stressed. The response scale is an ordinal 5-point scale, but the scores are grouped into a dichotomous variable for evaluating the items, being “Stressed” (Very Stressed and Stressed) and “Other” (Neutral, Moderately Stressed and Not Stressed). Thus, the lower the score, the more stressful the situation will be, and there is no cutoff point and no calculation of the score for each domain or total^(17)^.

The translation of the Student Nurse Stressor-15 (SNS-15) Scale, originally available in English, was carried out by two independent translators fluent in the language, one bilingual native (T1) and one sworn translator (T2), which resulted in two translated versions. It is noteworthy that the creator of the original version of the instrument authorized the process of translation, adaptation and validation of the SNS-15 for the Brazilian context in June 2021.

#### Synthesis

After the translations were carried out, the material was synthesized, through consensus between the researchers involved and a third native English translator who resides in Brazil. Thus, a common version was produced.

#### Back translation

The version resulting from the synthesis stage was sent to the two translators who participated in the translation stage, for back-translation purposes. The two back-translated versions (BT1 and BT2) were analyzed and, after consensus between the researchers and the translator who participated in the synthesis stage, a final version was obtained, which was sent to the main author of the instrument. At the time of this contact, clarifications were requested on items 1 “Preceptor”, 2 “Staff” and 9 “Staffing” of the SNS-15, in order to obtain the conceptualization used in the Irish version.

#### Review by a committee of experts

To form the committee of experts, the selection was made through analysis of the curriculum available on the Lattes Platform, administered by the National Council for Scientific and Technological Development (CNPq).

The population was made up of teachers with nursing training, linked to Higher Education Institutions. To this end, the eligibility criteria were adapted based on a model^(22)^: being a doctor; having active research and extension projects in the area of adult/elderly health; having scientific articles published in the area of adult/elderly health; acting in the clinical supervision of nursing students for a period of more than 2 years; and being linked to a group registered in the CNPq Directory of Research Groups in Brazil. The sample followed the recommendation of six to ten participants^(23)^.

Initially, a consultation was carried out in the CNPq Directory of Research Groups in Brazil, in order to locate nursing research groups focusing on the area of adult/elderly health. Then, the selected researchers were located through the “search resume” option, available on the main page of the Lattes Platform, to check the eligibility criteria.

The invitation was sent by email, individually, to each of the experts. They received the research information along with an access link to Google Forms, which contained the Free and Informed Consent Form (TCLE) for the specialist to indicate and confirm their participation in the research, if interested. Next, each specialist responded to a sociodemographic characterization form with the following variables: gender, date of birth, highest academic degree, post-doctoral internship, institution of affiliation, length of professional practice, position at the institution, participation in research group(s) in the adult/elderly area.

At the end, each expert filled out the face and content evaluation scale, in order to evaluate the semantic, idiomatic, cultural and conceptual equivalences between the original and translated versions of the SNS-15. Thus, when evaluating each item, the specialist had access to the term/expression from the Irish instrument (IE) and its version translated into the Brazilian context (BR), considering: -1 = Not equivalent, 0 = Undecided and +1 = Equivalent. A space was made available for observations that the evaluator considered relevant for each item evaluated. Those items that obtained at least 80% agreement between evaluators were accepted as equivalent items^(24)^. It is noteworthy that, among the members of the expert committee, some are proficient in the English language and the instrument validation method, as recommended by the adopted framework^(21)^.

#### Pre-test with nursing students

The pre-test was carried out with nursing students. Students aged 18 or over were included, regularly enrolled in one of the courses at the investigated institution and who were studying at least one discipline that included clinical practice activities, in order to maintain the context of development of the original version of the SNS-15. Students coming from international interinstitutional agreements and exchanges were excluded, due to nationality.

The pre-test sample followed the recommendation of 30 to 40 participants^(21)^. The pre-test was initially conducted remotely, via Google Meet, due to the transition from remote to in-person academic activities in the context of the COVID-19 pandemic. The snowball sampling technique was used. The first invitation to participate in this stage was made to a student linked to the researchers’ research group, who indicated the WhatsApp^®^ contact of the next possible participant, and so on. It should be noted that the first guest was not included in the sample to avoid conflict of interest. Thus, eight students agreed to participate in this stage.

Subsequently, the pre-test was conducted in person in the classroom. The convenience sampling technique was used. The students were approached by the researchers before classes began, at which time they were informed about the study. Those who agreed to participate received a data collection instrument and, after completing it, were given an opportunity to express doubts and provide suggestions. In-person collection was carried out with the participation of 24 students.

#### Assessment of psychometric properties

The evaluation of psychometric properties was carried out by a group of nursing students. The inclusion and exclusion criteria were the same as those adopted in the pre-test. To define the sample, a minimum of 10 participants per scale item^(25)^ was considered, totaling at least 150 nursing students, to carry out the factor analyses.

All students who were eligible, considering the inclusion and exclusion criteria, were approached before or after theoretical classes, at which time the research objectives were presented and the invitation to participate in the study was made. Thus, 238 nursing students participated in the evaluation.

#### Analysis of results and statistics

The data were organized and double-entered into an Excel^®^ spreadsheet to check their consistency and, after coding, they were exported for analysis in the IBM^®^ SPSS^®^ Statistics version 25 program, which allowed descriptive analysis.

To verify the feasibility of the factor analysis of the Brazilian version of the Student Nurse Stressor - 15 (SNS-15) Scale, Bartlett’s sphericity test was performed. To verify the number of factors in the Exploratory Factor Analysis (EFA), parallel analysis and the Scree Plot graph were carried out. In this case, an oblique rotation was used in the R program version 4.2.2, using the “psych” package; which allowed analyzing the correlation between the factors.

To verify the adjustments of the model of the Brazilian version of the Student Nurse Stressor - 15 (SNS-15) Scale, a Confirmatory Factor Analysis (CFA) was carried out using the “Lavaan” package in the R program version 4.2.2, developed for modeling latent variables. The following indices were considered: Comparative Fit Index (CFI), Tucker-Lewis Index (TLI), Root Mean Square Error of Approximation (RMSEA) and Standardized Root Mean Square Residual (SRMR). Reference values were adopted for CFI and TLI ≥ 0.90, RMSEA ≤ 0.08 and SRMR ≤ 0.10(25). Internal consistency was estimated considering the McDonald’s Omega coefficient (ω)(26), considering values above 0.7 as adequate^(27)^.

## RESULTS

In the version translated into Brazilian Portuguese, items 2, 9 and 10 were changed for better understanding in this context. With regard to items 2 - “Treatment by staff” and 9 - “Staffing levels”, a rapport was introduced in order to detail the composition of the “team” (comprising the nursing team and the health team). Regarding item 10 - “Clinical Placement Coordinator (CPC) relationships”, we chose to describe the acronym in order to facilitate understanding. All changes were approved by the lead author of the Student Nurse Stressor-15 (SNS-15) Scale.

The committee was made up of seven experts to evaluate the semantic, idiomatic, cultural and conceptual equivalences between the Irish version and the Brazilian version. Their sociodemographic profile is shown in Table 1.

**Table 1:** Sociodemographic characterization of the members of the expert committee (N=7), Ribeirão Preto, São Paulo, Brazil, 2022

Variables	n (%)
Gender	
Female	6 (85.7)
Male	1 (14.3)
**Highest academic degree (stricto sensu)**	
PhD	7 (100.0)
Completed post-doctoral internship	
Yes	1 (14.3)
No	6 (85.7)
Location of the institution to which you are linked	
North	1 (14.3)
North East	2 (28.5)
Midwest	1 (14.3)
Federal District	1 (14.3)
Southeast	1 (14.3)
South	1 (14.3)
Time working at the institution (years)	
2	1 (14.3)
3	1 (14.3)
4	2 (28.6)
9	2 (28.6)
10	1 (14.3)
Current position	
Adjunct professor or doctoral professor	7 (100.0)
Number of research groups in the adult/elderly area	
1	5 (71.4)
2	2 (28.6)

The items on the Irish scale 1 - “Preceptor relationships”, 6 - “Placement workload” and 13 - “Facilities e.g. canteen” obtained a Content Validity Index (I-CVI) equivalent to 0.57, which is why changes were made taking into consideration the suggestions of committee members. Among the changes, in addition to maintaining “preceptor”, the term “supervisor” was added to the first item. In the sixth item, the most recurrent suggestion was the addition of the expression “clinical practice”, which was accepted. In the thirteenth item, the expression “place to eat” was attributed, since all the recommendations made by the experts were regional terms, that is, they designated the same idea with different terminologies. When considering the 15 items of the Brazilian version of the Student Nurse Stressor - 15 (SNS-15) Scale, the global Content Validity Index (S-CVI) was 0.81.

Table 2 describes the items of the Student Nurse Stressor-15 (SNS-15) Scale, the version analyzed by experts, with their respective I-IVC and S-IVC; as well as the Brazilian version of the Student Nurse Stressor - 15 (SNS-15) Scale.

**Table 2:** Content Validity Index per item and overall version analyzed by experts (n=7) and the Brazilian version of the Student Nurse Stressor - 15 (SNS-15) Scale, Ribeirão Preto, São Paulo, Brazil, 2022

Item	The Student Nurse Stressor-15 (SNS-15) Scale	Version analyzed by the expert committee	CVI^*^	S-IVC†	Brazilian version of the Student Nurse Stressor - 15 (SNS-15) Scale
				0.81	
1	Preceptor relationships	*Relacionamentos com supervisores*	0.57		*Relacionamento com enfermeiro supervisor/preceptor*
2	Treatment by staff	*Tratamento pela equipe (Equipe de Enfermagem e Equipe em Saúde)*	0.86		*Tratamento pela equipe‡*
3	Clinical skills	*Habilidades clínicas*	1.00		*Habilidades Clínicas*
4	Medications	*Medicação*	1.00		*Medicação*
5	Being prepared	*Sentir-se preparado*	1.00		*Sentir-se preparado*
6	Placement workload	*Carga de trabalho do estágio*	0.57		*Carga de trabalho da prática clínica*
7	Academic workload	*Carga de trabalho* *acadêmico*	1.00		*Carga de trabalho acadêmico*
8	Resources e.g. equipment	*Recursos, por exemplo,* *equipamentos*	0.86		*Recursos, por exemplo, equipamentos*
9	Staffing levels	*Dimensionamento da equipe (Equipe de Enfermagem e Equipe em Saúde)*	0.86		*Dimensionamento da Equipe‡*
10	Clinical Placement Coordinator (CPC) relationships	*Relacionamento com o coordenador do estágio clínico*	0.71		*Relacionamento com o coordenador da prática clínica*
11	Patient/client relationships	*Relacionamentos com pacientes/clientes*	0.86		*Relacionamentos com pacientes/clientes*
12	Number of work days per week	*Número de dias de estágio por semana*	0.86		*Número de dias de prática clínica por semana*
13	Facilities e.g. canteen	*Facilidades, por exemplo, cantina*	0.57		*Facilidades, por exemplo, local para refeição*
14	Missing days on placement	*Faltas no estágio*	0.71		*Faltas no campo de prática clínica*
15	Length of journey to placement	*Tempo de deslocamento até o campo de estágio*	0.86		*Tempo de deslocamento até o campo de prática clínica*

* *CVI - Content Validity Index for each item; †S-CVI - Global Content Validity Index; ‡Equipe - Nursing Team and Health Team.*

32 nursing students with an average age of 22.4 years participated in the pre-test. Of these, 31 (96.9%) were female, 19 (59.4%) were studying Bachelor’s and Bachelor’s degrees, with 9 (28.1%) in the first year, 8 (25.0%) in the second year, 5 (15.6%) in the third year, 6 (18.8%) in the fourth year and 4 (12.5%) in the fifth year. It should be noted that an opportunity was provided to express impressions about the instrument, and analysis of its structure, in addition to the response time and proportion of missing data. This step was fundamental in directing data collection, as it provided information about the estimated response time and possible doubts that could arise during this process. The instrument was well accepted and understood by students and therefore no changes to its structure were necessary.

Table 3 presents the results of the sociodemographic characterization of the 238 students who participated in the psychometric properties assessment stage. It should be noted that the participants included in the pre-test did not make up the sample.

**Table 3:** Sociodemographic characteristics of the nursing students (N=238), Ribeirão Preto, São Paulo, Brazil, 2022

Variable	n (%)	Mean	Median	Standard deviation	Minimum - maximum
Gender					
Female	213 (89.5)				
Male	25 (10.5)				
Age (years)		22.3	22.0	3.6	18-43
18 to 25	206 (87.7)				
> 25	29 (12.5)				
Course					
Bachelor’s degree	109 (45.8)				
Bachelor’s degree and teaching license	129 (54.2)				
Current year in the course					
First	67 (28.2)				
Second	64 (26.9)				
Third	36 (15.1)				
Fourth	40 (16.8)				
Fifth	31 (13.0)				

It is noteworthy that the p-value for the Bartlett test was less than 5%, which indicates the usefulness of the factor analysis. The parallel analysis indicated the extraction of four factors, unlike the original version of the SNS-15, which has two factors. Table 4 presents the factor matrix of the Brazilian version of the Student Nurse Stressor - 15 (SNS-15) Scale, detailing the four factors and the psychometric parameters estimated for the items.

**Table 4:** Factor matrix of the Brazilian version of the Student Nurse Stressor - 15 (SNS-15) Scale (N=238), Ribeirão Preto, São Paulo, Brazil, 2023

Item	Factor loading value 1	Factor loading value 2	Factor loading value 3	Factor loading value 4
3	**0.93**	-0.06	0.06	-0.04
4	**0.71**	0.08	-0.04	0.02
5	**0.67**	0.07	-0.09	0.08
12	0.11	**0.62**	0.18	-0.08
13	-0.07	**0.59**	-0.07	0.01
15	-0.09	**0.58**	-0.05	0.07
6	0.24	**0.44**	0.05	0.10
14	0.08	**0.43**	0.21	0.02
11	0.13	**0.23**	0.14	0.14
1	0.02	0.02	**0.80**	0.02
2	0.00	-0.02	**0.61**	0.05
10	-0.06	0.19	**0.42**	0.19
9	0.01	-0.11	0.06	**0.71**
8	-0.04	0.16	0.06	**0.56**
7	0.14	0.32	-0.19	**0.32**

The items have a good correlation with the extracted factors, unlike item 11, which had a low factor loading. Despite this, it was decided to maintain this item, upon consensus with the researcher who developed the instrument. In the end, the items were reorganized among the four factors, which gave rise to the Brazilian version of the Student Nurse Stressor - 15 (SNS-15) Scale.

Through Confirmatory Factor Analysis of the Brazilian version of the Student Nurse Stressor - 15 (SNS-15) Scale, the following satisfactory fit indices were obtained (CFI = 0.94; TLI = 0.93; RMSEA = 0.06; SRMR = 0.16) and McDonald’s Omega coefficient (ω) obtained a value equivalent to 0.86.

## DISCUSSION

Although there are instruments validated in Brazil designed to assess the stress of nursing students^(28-30)^, none have the objective of identifying the stressors experienced by these students during their clinical activities with the elderly population. In this way, the Brazilian version of the Student Nurse Stressor - 15 (SNS-15) Scale greatly contributes to the improvement of nursing education from the perspective of care for elderly people, especially when considering population aging^(31)^. Considering the fact that the original version was validated with nursing students on their clinical placements with the elderly, and that there are no specific and exclusive fields for this population in the Brazilian institution where this research was conducted, the validation of this instrument with students who work in other specificities is justified. Based on this understanding, the cultural adaptation and evaluation of the psychometric properties of the Student Nurse Stressor-15 (SNS-15) Scale for Brazil were carried out.

Indeed, Brazil has cultural differences that become even more evident when considering the particularities of its regions, especially linguistic diversity^(32)^. Thus, from the beginning of this study’s design, it was envisioned that the expert committee would be composed of researchers from all five regions and the Federal District. This goal was achieved and, although disagreements in terms and expressions were noted, it is clear that the instrument obtained a CVI of 0.81, highlighting satisfactory agreement in the analysis of the original version adapted for Brazil^(24)^.

In the pre-test, the recommendations of the proposed framework were met^(21)^, and the Brazilian version of the Student Nurse Stressor-15 (SNS-15) Scale was well understood by the majority of participants. Only item 9 “Team sizing” was the one with the least understanding. It is believed that this is justified by the fact that discussions about staffing occur in the final periods of nursing courses.

Exploratory Factor Analysis (EFA) showed that four factors are necessary for the Brazilian version of the Student Nurse Stressor-15 (SNS-15) Scale. Furthermore, with the exception of item 11, all items obtained factor loadings greater than 0.30, which is the recommended value for samples of less than 300 participants^(25)^, as in this study. Therefore, the following names were assigned to each factor, namely: Self-perception in the clinical practice environment (3 - Clinical Skills; 4 - Medication; 5 - Feeling prepared), Dynamics in the clinical practice environment (6 - Load working hours of clinical practice; 11 - Relationships with patients/clients; 12 - Number of days of clinical practice per week; 13 - Facilities, for example, place to eat; 14 - Absences in the field of clinical practice; 15 - Commuting time to the field of clinical practice); Interpersonal relationship with the professional team (1 - Relationship with supervisor/preceptor nurse; 2 - Treatment by the team*; 10 - Relationship with the clinical practice coordinator) and Demand and theoretical-practical knowledge (7 - Academic workload; 8 - Resources, for example, equipment; 9 - Team sizing*). It should be noted that, to name the title of each factor, the item that obtained the highest factor loading in each factor was considered.

The Confirmatory Factor Analysis (CFA) of the Brazilian version of the Student Nurse Stressor-15 (SNS-15) Scale showed good adjustment indices (CFI = 0.94; TLI = 0.93; RMSEA = 0.06; SRMR = 0. 16), which are close to the values found in the original version of the SNS-15 (CFI = 0.93; TLI = 0.92; RMSEA = 0.06)^(17)^. This information highlights the good suitability of the model obtained by Exploratory Factor Analysis (EFA). It is also noteworthy that the McDonald’s Omega coefficient (ω) obtained a value equivalent to 0.86, which indicates good reliability of the instrument. Consequently, the Brazilian version of the SNS-15 proved to be a valid scale for use with students undertaking all types of clinical internships.

It is clear that the advent of the COVID-19 pandemic represented a challenging context for nursing education, highlighted by research conducted during the pandemic period that indicates that nursing students were more stressed due to the difficulties encountered in the teaching and learning process^(33-35)^.

### Study limitations

A limitation of the study is the use of a three-point scale to assess agreement between the members of the expert committee; however, it is emphasized that in the literature there is no consensus on the number of points to evaluate the CVI. Furthermore, the test-retest was not performed, making it impossible to analyze the stability of the instrument over a short period.

### Contributions to the field of Nursing

The instrument provides a valid assessment tool for nursing education, that contributes to the training process of students, especially when considering the transformations and stressors related to the period of the COVID-19 pandemic, such as the abrupt transition from in-person teaching to remote and the resumption of activities in a hybrid or in-person format, among others.

## CONCLUSIONS

The Brazilian version of the Student Nurse Stressor - 15 (SNS-15) Scale presents semantic, idiomatic, cultural, and conceptual equivalences with the Irish version, while its evidence confirms its validity and reliability in identifying stressors of Brazilian nursing students in the clinical practice environment. Thus, the scale can be used in the clinical education of nursing students, in order to support the development of investigations and interventions aimed at improving the teaching-learning process, and also students’ emotional safety and well-being.
